# Neuronavigated Transcranial Pulse Stimulation (TPS) with shock waves as a novel tool of noninvasive brainstimulation (NIB) for a long-term treatment of Alzheimer’s disease

**DOI:** 10.1192/j.eurpsy.2025.558

**Published:** 2025-08-26

**Authors:** U. Sprick, A. R. Günes, M. V. Beglau, M. Köhne

**Affiliations:** 1 Center of Neurostimulation; 2 Alexius/Josef Clinic, Neuss, Germany

## Abstract

**Introduction:**

Alzheimer’s disease (AD) is a very common cause of dementia and a common cause of death in elderly humans. No effective long-term treatment has been found so far. Recently developed treatments with antibodies have shown severe side effects of edema or intracerebral hemorrhage in a larger number of cases.

**Objectives:**

Neuronavigated transcranial pulse stimulation (TPS) as a new non-invasive therapy method could represent a current alternative to standard treatments. In contrast to ultrasound stimulation (tFUS) TPS uses shock waves with a mechanical transduction. These shock waves allow an application to superficial brain structures as well as into areas deep in the brain without the induction of any unwanted thermal side effects. The stimulation of the target areas can be MRI-navigated with nearly a similar precision as in stereotactical procedures.

**Methods:**

85 out-patients with Alzheimer’s disease with light to moderate symptoms received TPS-treatments with 6.000 pulses each session bilaterally in 6 sessions over 2 weeks into the frontal, parietal and temporal cortex (0.2 mJ/mm^2^ 4 Hz - Neurolith by Storz Medical). The treatment was repeated with a single booster session every 6 weeks. Pulses were inividually neuronavigated by current MRI-images. Executive functions were tested using the Stroop-Test (colour-word-interference-test). Patients with Alzheimer’s Disease normaly present only poor results in the Stroop-Test. We tested with a pre – post design (t_0_ pre stimulation : t_1_ after 6 sessions, two weeks later as well as t_2_ 6 months later). The mood of the patients was measured using the BDI on t_0_, t_1_ and t_2._

**Results:**

TPS-stimulation showed strong ameliorating effects on performance in the Stroop-Test. The mean-score of the Stroop-test was diminished significantly (pre vs. post ; p < 0.05 – paired T-test) in a comparison of t_0_ to t_1_. This effect was preserved during an interval of 6 months (t_2_). Single patients showed extraordinary improvements by shortening completer times in the Stroop-Test by half.

Depressive symptoms of the patients were also diminished by the treatment. The BDI score decreased from 20.1 ( t_0_ ) to 9.7 ( t_1_), and 9,1 (t_2_) respectively.

No significant side-effects occured during all the sessions in any of the patients.

**Conclusions:**

The results of this trial show that cognitive impairments of executive functions and depressive symptoms in Alzheimer’s disease may be ameliorated using TPS as a noninvasive neuronavigated brain stimulation method. No severe side-effects were observed. In the meantime beneficial effects of shockwaves with low intensitiy have also been shown in the fields of dermatology, orthopedics and cardial surgery.

Different mechanisms of action of TPS are still under investigation.

**Disclosure of Interest:**

None Declared

